# “Sometimes I don’t have a pulse … and I’m still alive!” Interviews with healthcare professionals to explore their experiences of and views on population-based digital health technologies

**DOI:** 10.1177/20552076211018366

**Published:** 2021-05-22

**Authors:** Flavio Tomasella, Heather May Morgan

**Affiliations:** 1 School of Medicine, Medical Sciences and Nutrition, University of Aberdeen, Aberdeen, UK; 2 Institute of Applied Health Sciences, University of Aberdeen, Aberdeen, UK

**Keywords:** Digital health, mHealth, wearables, smartwatches, smartphones, health apps, self-tracking, healthcare professionals, qualitative interviews

## Abstract

**Background:**

Digital technologies are increasingly becoming an integral part of our daily routine and professional lives, and the healthcare field is no exception. Commercially available digital health technologies (DHTs – e.g. smartphones, smartwatches and apps) may hold significant potential in healthcare upon successful and constructive implementation. Literature on the topic is split between enthusiasm associated with potential benefits and concerns around privacy, reliability and overall effectiveness. However, little is known about what healthcare professionals (HCPs) have experienced so far with patients and what they perceive as the main advantages and disadvantages of adoption. This study therefore aims to investigate current perceptions of HCPs towards self-tracked health-related outputs from devices and apps available to the public.

**Methods:**

Nine HCPs volunteered to take part in semi-structured interviews. Related data were thematically analysed, following a deductive approach with the construction of a framework based on expected themes from the relevant literature, and themes identified from the first two interviews.

**Findings:**

The following main themes in relation to DHTs were identified and explored in detail: HCPs’ experience, knowledge and views; advantages and disadvantages; barriers towards healthcare implementation and potential solutions; future directions. While most participants were adopters of DHTs and held positive views about them, their overall experience with patients and the technology was limited. Potential reasons for this were explored, including factors such as time/resources; colleagues’ mindset; lack of evidence of effectiveness for practice; data security concerns.

**Conclusions:**

The potential advantages of DHTs’ adoption in healthcare are substantial, e.g. patient autonomy, time/resources saving, health and behaviour change promotion, but are presently premature. Therefore, future research is warranted, focussing on addressing barriers, minimising disadvantages, and assessing the clinical value of commercially available DHTs.

## Background

Digital technologies are increasingly becoming an integral part of our daily routine and professional lives, and the healthcare field is no exclusion. Their use and integration in clinical settings is well documented and increasingly advocated in the literature.^1^,^2^ Recent research found that the number of health-related apps available to the general public on major app stores worldwide was over 318,000 in 2018, which is nearly double what was available just three years earlier (2015), and the overall trend suggests further increases in numbers, diversity and features.^3^,^4^ These figures are very much in line with the progressive increase of smartphone ownership worldwide, particularly in the US and the UK.^4^

In this context, it is therefore unsurprising that the WHO has very recently (April 2019) released the first version of evidence-based guidelines on digital health interventions,^5^ committing to issue continuous updates to the document as evidence progressively emerges from research. Similarly, at a UK national level, the NHS is currently working on a series of digital health strategies to improve the overall healthcare service, sustainably, such as improved and more secure patient data handling and going progressively ‘paperless’ by 2020.^6^

It has long been established that the leading causes of death, globally, relate to cardiovascular events – 31.7% of all deaths (17.9 million) annually.^7^ Thus, it is no surprise that apps and devices specifically targeting cardiovascular health are prominent; a key example and recent advancement in this field is the latest Apple Watch®, able to perform simple ECG tests.^8^

Recent studies have explored the potential implications of ‘mHealth’ (i.e. mobile health)^9^ integration, or ‘gap bridging’ between the personal and clinical/professional fronts, with quantitative data gathering, e.g. surveys.^10–12^ From these, awareness of the potential benefits (e.g. better and more frequent health parameters monitoring and tailoring of related interventions) and issues (e.g. lack of validity, reliability and scientific evidence of effectiveness) associated with commercially available mHealth technologies, referred to as digital health technologies (DHTs) throughout this paper, is evident. However, much less is known about what healthcare professionals have experienced so far in their everyday practice and think in this regard – e.g. their personal or professional encounters, what their views and thoughts are, and whether there are any barriers towards successful implementation of DHTs in healthcare practice that need to be identified and resolved. Indeed, along with technological innovation and general enthusiasm around DHTs, there is also, on one hand, growing concern around potential issues such as imprecision, lack of reliability and panic-inducing false-positive diagnosis.^8^ On the other hand, however, there is a perceived potential for missed opportunities,^13^ due to barriers towards a constructive and more effective implementation and use of these technologies by front line healthcare professionals (HCPs), such as general practitioners (GPs) and community pharmacists (CPs).

Recent qualitative work has further attempted to shed some light on the current phenomenon exploring healthcare professionals’ attitudes towards the adoption of digital/wearable technologies by the public for health care/tracking.^14^ The study findings show a significant level of scepticism among HCPs about the use of DHTs, though the focus of the study revolved almost exclusively around attitudes and perceptions, rather than experiences. Indeed, much less is known about what HCPs find the main disadvantages, barriers and related solutions, to be towards constructive implementation of DHTs in healthcare.

Some qualitative research has been conducted into the use of DHTs with patients who have chronic conditions, such as diabetes, obesity, Chronic Obstructive Pulmonary Disorder (COPD) and cancer.^15–20^ Many of these studies have focused on ‘end user’ or patient experiences and the potential of DHTs. When they have included HCP participation among the samples, the research concern has still been that of the patient. There has been little attention paid to HCPs’ own experiences, but more on speculation about perceived additional workload^21^ and the need for education and training.^22^ In relation to cardiovascular (CV) disease, scoping reviews have been carried out regarding DHTs,^23^ as well as commentaries^24^ on their potential or scientific statements^25^ about self-care, which may include DHTs, but we could identify no qualitative studies of HCPs’ perceptions towards self-tracked CV health-related outputs.

To gain insights into the growing digital ‘health tech’ phenomenon, and to understand it from the perspectives of professionals involved, the present study followed a qualitative approach, with the aim of investigating current perceptions of HCPs towards self-tracked CV health-related outputs from devices and apps available to the public, to further the existing literature and inform practice, given the gap in knowledge around HCPs’ experiences. This was achieved by:

be achieved by:

- exploring current experiences of HCPs in dealing with patients or clients concerned about their wearable device’s, CV health-related, output, and perceived barriers and disadvantages to their use in healthcare practice, as well as related solutions;
- evaluating HCPs’ perceived preparedness in dealing with issues and concerns from the general public related to digital, self-monitored CV health parameters, e.g. blood pressure (BP) and heart rate (HR);
- identifying potential gaps in the research around HCPs’ knowledge, awareness and ability to deal with the rapidly increasing popularity of self-monitoring apps for health and lifestyle, to be explored in future research.

## Methods

### Population of interest and recruitment strategy

The population of interest for this study comprised front line healthcare professionals (GPs, CPs and nurses) as well as professionals in training (medical students), intended to be sampled until achievement of data saturation and richness and quality of data.^26^ No exclusion criteria were applied to this population, nor any geographical restrictions for recruitment.

**Figure 1.**
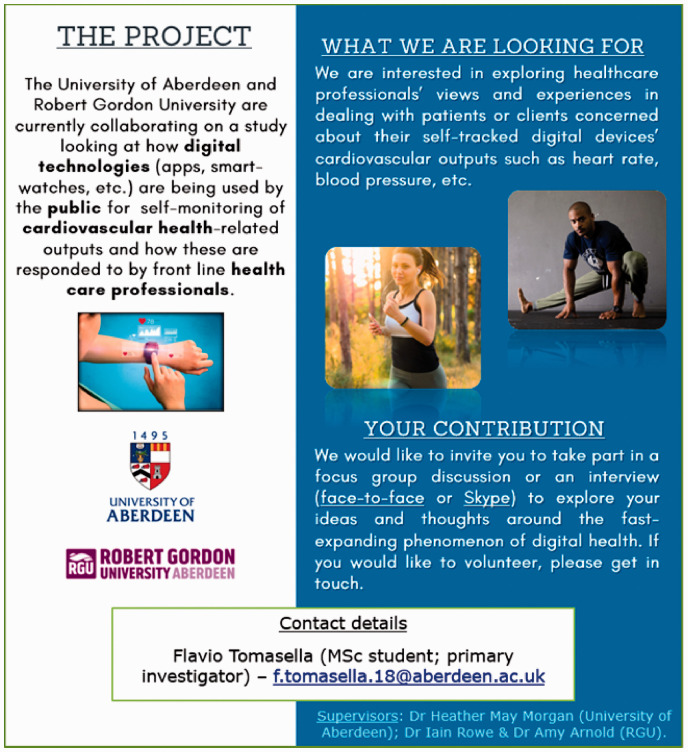
Recruitment advert.

Participants were recruited via personal and professional networks of the research team, by advertising the study on social media (professional groups, hashtags, etc. on Facebook, Twitter, and LinkedIn), with the aid of an advert (see Figure 1). Paper copies of the advert were also used to advertise the study, locally, within academic premises (Robert Gordon University and University of Aberdeen), for snowball sampling purposes, and at a conference on digital health strategies in Newcastle – Digital Catalyst 2019, an event which convened a mix of participants including researchers, HCPs and potential users of research outcomes to discuss the current progress of DHTs and the future challenges that need to be addressed.^27^

### Data collection

Data were collected between May and July 2019 in the form of digital audio files during the conduct of semi-structured one-to-one in-depth interviews^28^ undertaken, either face-to-face or by telephone/Skype, with the aid of a topic guide. Digitally recorded audio files from the interviews were then manually transcribed verbatim by FT, on ‘.docx’ files with the aid of the pedal-operated software Express Scribe Pro (NCH Software Inc. Version V8.06, 2019),^29^ then loaded on NVivo (QSR international Pty Ltd. Version 12, 2019)^30^ for subsequent data analysis. As per protocol, focus groups (FGs) were also to be conducted for the purpose of data collection, if it were possible to bring together more than one HCP at a time, aiming for heterogeneity of participants within each of them in order to stimulate discussion around subjective views and/or debatable aspects of the topic.^31^,^32^

### Data analysis

Data analysis took place alongside the data collection process on a framework created in parallel by FT and HMM, based on expected themes determined from the literature, and those that were identified in the first two interviews. The few discrepancies between the two versions were then resolved through discussion. Appendix 1 shows the final framework adopted for data analysis. NVivo data analysis was conducted by FT, and then **reviewed** by HMM. Potential discrepancies were resolved through discussion in this occasion as well.

Specifically, data were analysed according to the Ritchie and Lewis method for thematic analysis.^33^ Upon familiarisation with the transcripts, codes were created and themes were identified and built into the framework that took into account those determined from the literature. The framework was well developed after the first two interviews, with additional codes and themes being added as necessary from subsequent transcripts. Codes and themes were periodically reviewed for quality throughout the data collection process.

**Figure 2.**
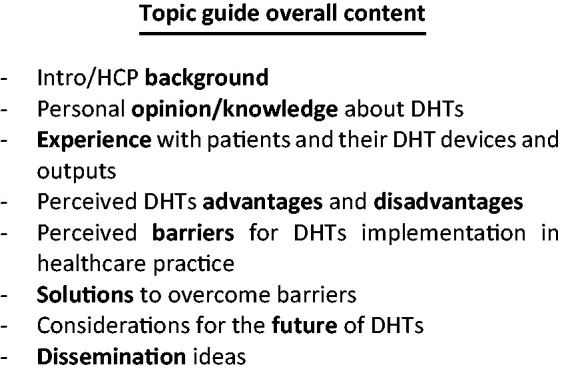
Topic guide indicative content.

The data analysis conducted in tandem with data collection allowed for the necessary amendments to the topic guide prior to conducting subsequent interviews, which was then partly based on either sufficient coverage of certain themes and aspects or, conversely, the need to expand more systematically on newly emerged themes. Figure 2 shows the indicative content of the topic guide. Given the semi-structured nature of the sessions, some questions were asked outwith the topic-guide schedule, based on spontaneous conversation turns, with the intention of probing and stimulating further contextual insight. The topic guide was designed prior to any interview being conducted, based on findings from previous literature. As interviews took place and data was gathered, the related findings were used to inform necessary changes that would ensure richness of data collection.

This study was reviewed by the Ethics Review Board of the College of Life Sciences and Medicine of the University of Aberdeen (Application No. CERB/2019/4/1765 – approved 24 April 2019). All participants gave written, informed, consent for participation and publication of quotes.

## Findings

### Sample

A total of n = 9 participants were recruited via convenience and snowball sampling (see Table 1 for a summary of sample characteristics and labelling used for quotations). Of the n = 8 individuals contacted and invited directly to take part in the study, 50% (n = 4) did partake. Overall, the sample comprised: n = 5 medical professionals (n = 3 GPs and n = 2 medical students); n = 3 nursing staff (n = 2 registered nurses and n = 1 auxiliary nurse/clinical change assistant for NHS Digital) and n = 1 community pharmacist/academic lecturer. The age span of the sample was between mid-twenties and late fifties, all but two from the UK. The single participant from the US was recruited as a personal contact, a medical doctor expert and actively involved in global health action, invited to take part in the study due to the worldwide relevance of cardiovascular diseases, and to gain some insight into DHTs in terms of practicality and feasibility of their implementation in settings other than a high-income country, such as the UK. All but two interviews were conducted face-to-face. One interview was conducted over the phone (participant 008) and another one via Skype (participant 007).

**Table 1.** Participants details.

Participant number	Role as HCP	Quotation labels	Nationality
001	Pharmacist	Pharmacist	UK
002	Medical student (4th year)	Med student	UK
003	GP	GP and academic researcher	UK
004	Medical student (junior doctor)	Med graduate	UK
005	GP	GP and academic lecturer	UK
006	Nurse	Nurse	Greece
007	GP and global health (GH) expert	GH expert	US
008	Auxiliary nurse and NHS digital employee	Aux nurse	UK
009	Nurse	Nurse and academic	UK

As mentioned, FGs were also supposed to be conducted. However, despite a successful outreach of the advert through social media (one tweet managed to get over 1,500 impressions), the steady-but-slow progression of participant enrolment to the study meant there was never a sufficient number of participants available at once to run focus groups during the study’s data collection phases.

### HCPs familiarity, experience and perceived knowledge with DHTs

Overall, most participants did have some familiarity with DHTs for personal use, primarily apps and wearable devices, for a variety of health-related purposes and tracking – sleep, running, heart rate monitoring, mindfulness, etc.

*‘I have used FitBits before. I’ve also used My Fitness Pal, but that was mainly just for, like, recording what I’ve eaten so  …  yeah, and I find them really useful actually for monitoring … ’* [Med Student].

*‘Yeah, I mean, I am familiar with the apps. I think self-monitoring’s one of the main components of health apps. I guess I kind of think of the difference between fitness trackers, cause a lot of people wear fitness trackers, and then apps that people have downloaded to deal with a specific condition.’* [GP and Academic Researcher].

*‘I’m using a physio app myself called Reach – you generally need it for about 10 weeks; you fill it in – it asks loads of questions about you, pain, type of pain and where it is – and then they device some exercises for you and for those exercises, there’s a video for you to watch so you can replay it’* [Nurse and Academic].

*‘Uhm … what else am I using … ? I think Headspace, you know, for like meditation … sometimes I use the ‘Heath’ app for like counting steps but probably use my watch a bit more for that*’ [GP and Academic Lecturer].

However, most of them also had very few experiences of dealing with patients wishing to discuss their cardiovascular health status based on outputs from their own devices and apps.

*‘So, yeah, we’re not really knowing about that, we’re not really promoting it and when I’m saying, earlier in the interview, that there’s a lot of problems like weight management, obesity and things, you know, actually, there’s probably huge scope for us to be embracing this a bit more! Uhm, but I guess it’s also new, isn’t it? Like, we don’t have the evidence – it’s not becoming part of popular practice or culture’* [GP and Academic Lecturer].

The med students also stated that digital heath technologies are not very much, if at all, part of their training programme.

*‘[I am] only vaguely [familiar with digital health apps and devices], but I couldn’t think of any specific examples or anything so I think there’s a lot out there that I’m not aware of and probably should be more aware of but it’s not something that is in our curriculum or anything like that so … ’* [Med Student].

Although personal use of digital health tech was quite popular among the participants, their experience as HCPs dealing with patients presenting with similar data was, sometimes to their own surprise, very modest.

*‘I’m thinking about my job as a clinician … I actually am quite surprised that I haven’t had more patients come to me with problems or issues [associated with digital readings/outputs]’* [GP and Academic Lecturer].

*‘I’ve had this maybe 2 or 3 times. So, my patients tend to be […] quite an affluent group. […] And a lot of my patients, hardly any of them smoke, a lot of them do exercise, uhm, cardiovascular disease isn’t one of the main problems in the practice and a lot of people take in active uhm interest in their cardiovascular health. […] And a lot of them do wear smartwatches […]. And I’ve had a couple of scenarios where they’ve got unexpected HR readings from them? […]. [I] had one gentleman who was concerned about his HR readings on his watch and … I can’t remember actually if he was too fast or too slow […]. One of my colleagues had referred him to cardiology, and then he made the decision, on his own, that actually he was so well … and all those tests in the practice, ECG and heart rate, had been fine, that he decided not to go to the cardiology appointment’* [GP and Academic Researcher].

A main reason provided as to why the participants’ experience with patients and their digital health gear was limited, despite the evident proliferation on the market and personal ownership, was the fact that, overall, the phenomenon is perceived to be at early stages.

*‘It’s quite an emerging area of practice that we haven’t quite caught up with’* [GP and Academic Researcher].

‘*Come to think of it, I’m highly surprised that that is not the case, and we’ve discussed already the reasons why – I wonder if there’s a bit of lag with this’ [i.e. between the technology being finally available, and patients/healthcare providers making ordinary use of it]*. [GP and Academic Lecturer].

*‘I think it’s just … a lot of digital technology’s still in its infancy?*’ [Med Student].

### HCPs attitudes towards patient-owned DHTs

The overall attitude towards patient-owned, or ‘commercial’, digital health devices and apps was largely positive, despite the significant lack of experience in dealing with it personally.

*‘It’s the way forward! And people need to stop saying ‘oh but I’m used to pen and paper’ well – tough! Cause pen and paper is inefficient and it’s taking you away from your patient – stop what you’re doing, pick up an iPad, or a computer or a laptop, and get on with it and with your patient! [This would result in] more time with your patient! Patient care is the beginning and the end of everything we do, so therefore that should be our focus!’* [Aux Nurse].

*‘I think it would be a useful tool to start conversations with healthcare professionals (HCPs) regarding results/health in general.’* [Pharmacist].

Only one participant felt that these technologies could, potentially, be more of an issue and hindrance to healthcare practice, than something beneficial.

*‘With the general public who probably don’t have that level of [medical/scientific] knowledge it’s really challenging and you need to provide a lot of background – you need to go right at the start and … we just don’t have time or capacity to do that. I think it would also lead to so much unnecessary anxiety and worry. […] Uhm, I think we need to just be really careful that the line is sitting on that side – that it’s a helpful thing for patients, and it doesn’t just become burdensome, it doesn’t become extra workload for doctors’* [GP and Academic Lecturer].

### Digital health and the online environment

Participants were also invited to reflect on digital health aspects in terms of information that patients may gather from a simple online search. Indeed, participants have often had experiences of seeing patients who had previously searched their symptoms online and then sought expert advice with a ‘self and pre-made’ diagnosis. Quite often, participants found the patients’ self-gathered information either to be the unrealistic extreme worst-case scenario of the correct condition or even the wrong one completely.

*‘The only disadvantage I would have would be somebody who sits at home and has all of these … like they go on the internet, they go on apps and self-diagnosis, where they could be completely wrong – I’ve seen it happening when I’ve done a shift on A&E’ [Aux nurse].*

*‘I think there’s an emphasis on serious worst-case scenario-thinking on searches’* [GP and Academic Researcher].

A recurring theme in this context was the participant concern about the reliability of the online source of health knowledge and how this can, sometimes, not only misinform but also cause distrust in patients towards their healthcare providers.

*‘I find that people do come with ideas about conditions or treatment that they have found online. Sometimes this is helpful, although not always. It is sometimes difficult to change opinions on some issues like antibiotics requested when not necessary’* [Pharmacist].

*‘I would say about, uhm, 20 to 25% of the time patients come with inaccurate information. […] I mean, Facebook is the devil when it comes to medical information – everybody thinks they’re an expert on Facebook, uhm, and same thing with these online blog where people form these communities and start talking about nonsense – like, they start talking about vaccines or ‘this drug is harmful’ ‘that drug is harmful’ without knowing any kind of evidence base behind that’* [GH Expert].

By contrast, one of the participants was, cautiously, more optimistic and trusting of their patients’ ability to be critical about the information gathered online.

*‘My gut feeling is that people are relatively sceptical about online resources – they sort of do a bit of scoping, to see what’s out there, and use it as part of their decision-making but not all of it and usually people are quite sensible about knowing that the internet is not a definitive and trustworthy source of information’* [GP and Academic Researcher].

Questions related to digital/online health also led to the understanding that in the UK the NHS has started a database of ‘approved apps’ with the aim of ‘helping patients and the public to find trusted health and wellbeing apps’, assessed by the NHS and deemed clinically safe.^34^

*‘Well, I think, if you’re going to be using apps in healthcare then you need to have some kind of “central library” of approved apps. And I think that’s what the NHS started doing. So they’ve got the “NHS apps library”, […] I think that’s a good kind of framework in that there’s people within the NHS assessing the suitability of apps and how accurate they are so then people can go online and see “oh this has been approved, so I’m happy to use this”’* [Med Student].

Meanwhile, the NHS has also started some initiatives aiming to move towards a ‘as digital as possible’ environment within their systems and hospitals, by a set deadline.

*‘[…] NHS digital – this team works around GDE, Global Digital Exemplar trust, from Newcastle. So we got £5 million from the government and we added another £5 million, which made this trust as digital as possible by the year 2020, […]. And what we need to do as part of the “NHS digital” focus is to make the NHS at least 80% paper-light, by 2020. We are never going to become completely paper-light because we always need business continuity plans. But that’s in all aspects, whether it’ll be cardiology, elderly care, mental health, medicine, surgery, everywhere needs to become more digital and also so we can then care with things like other trusts – GP practices, the community teams, etc … ’* [Aux Nurse].

### Reliability concerns

Concerns associated with overall reliability of the tech available to the general population (such as validity, accuracy, calibration), both in terms of intrinsic issues and limitations of the tech, as well as user error, were a theme which frequently emerged among the participants.

*‘I’m not sure what the [manufacturers’] standards are – you know, heart rate, for example, [or] pulse – I don’t know what they use to determine whether their devices are accurate or not. Because I know, from my use that, you know, sometimes I don’t have a pulse … and I’m still alive [laughter]. Something’s not quite right. And whether that’s the device itself, or the user – user error might contribute to some significant discrepancies between what the result actually is and what appears on screen’* [Pharmacist].

The current approach to solving the issue was, virtually across the board, to double-check the readings from the personal devices with gold standard and validated professional equipment.

*‘I would be wary of the readings obtained from these devices due to lack of calibration, reliability, potential for user error, however, may direct individuals to HCPs for measurements taken using calibrated equipment’* [Pharmacist].

*‘I think I’d be a little apprehensive, because I don’t know if it’s always accurate, I guess that it is, but, it’s subtle […] And if, like, they’re saying ‘oh my blood pressure’s like “this” on my smartwatch’ … I’d want to check it myself, rather than put my, like, approval on some piece of technology’* [Med Graduate].

One of the participants also provided an alternative explanation as to why there may be discrepancies in outputs/readings from a digital device and the actual or alleged health status of the individual using it, based on our current understanding of what’s ‘normal and healthy’, which may be limited, and consequent cut-off values.

‘*[…]and I said to [my patient concerned about self-monitored heart rate readings] “a) we don’t know if they’re 100% accurate … ”, you know, his clinical examinations had been fine; he was exercising at quite intensities without any symptoms “ … so you’re quite at low-risk”. The other thing that I questioned in my head is the way we’ve developed our reference ranges for things? Or ideas about “norms”? I wonder if there’s any time in the history of medicine when we’ve taken literally millions of healthy individuals and have measured their HRs over extended periods of days, months, years’* [GP and Academic Researcher].

### Perceived advantages of patient-owned DHTs

Participants were actively invited to consider potential advantages of DHTs available to the public. In this context, the advantages that emerged during conversation were: a) behaviour change; b) aid conversation with HCPs; c) patient autonomy/minimising the ‘white coat syndrome’/time and resources-saving.

- Behaviour Change

*i) ‘And I think you can encourage people! If you set small goals, like ‘oh you should walk six-thousand steps a day’ then if it’s achievable for that person, then they’ll want to do it, and then you can increase the goals’* [Med Graduate].

*ii) ‘It motivates you to get more active […]. And you know, if you do one lifestyle change, then other changes will start to follow. So if you start keeping track of your health, and it says that your HR is a bit high, so: you take actions to lower your heart rate; uhm, you may change your diet as well; you go to sleep earlier; you relax a bit more. You get more conscious about your general health and I think that at the end you learn to love yourself better, to respect yourself better’* [Nurse].

b. Aid conversation with HCPs

*‘I think it would be a useful tool to start conversations with healthcare professionals (HCPs) regarding results/health in general’* [Pharmacist].

c. Patient autonomy/minimising the ‘white coat syndrome’/time and resources-saving

*i) ‘I think it would be really useful, because I think there are only so many things you can monitor in a hospital […]. I think especially if you wanted to monitor things over a certain length of time, it’s much easier if the patient has control of that and they can do it themselves? So, it’s definitely something I would be open to listen and talk to the patient about’* [Med Student].

*ii) ‘I think [with this kind of tech] you can get measurements over a much greater time period and, as well, when people come into hospital […] they might get nervous so it’s difficult to insure you are getting accurate readings of things, whereas if you just give them something that they can track within their daily life, you know you’re getting a much more accurate representation, especially when it comes to things like cardiovascular heath, because with, obviously, if people are nervous, sometimes their BP can rise, so if you’re measuring that outside of a hospital setting, then you know it’s more accurate’* [Med Student].

With patient autonomy with health tracking, the potential consequent advantage of relieving some pressure on the healthcare system also emerged.

*iii) ‘But I think, overall, definitely, there’s a lot of potential and it could free up a lot of NHS time and resources, which is obviously a huge problem at the moment … ’* [Med Student].

One participant, based on a personal experience as a patient themselves, had particularly positive views in terms of patient autonomy and the ability to self-monitor and keep track of their own health at any time. The following quotation was inspired by a question about a recent smartwatch able to perform a single-lead ECG reading.

*iv) ‘I’ve got a heart murmur […] [and] abnormal heart rhythm, but only occasionally so if I had an ECG it would be fine because it wouldn’t be when the thing is happening. But I bought myself a heart monitor; it’s just a small thing and you hold it [between your hands sort of gesture] and it comes up with a small tracing [which the GP was happy about, though it couldn’t provide good or long enough readings]. […] And then I saw the watch … and that’s what I want for Christmas! Because […] then I can give my GP the data and then they can decide what needs to be done. So I know that my GP would be willing to take something like that because [we already had similar conversations]. But I obviously can’t just go in to the doctors because […] by the time I’ve got an appointment it [my ECG reading] would be OK again! […] And I’m not ill enough to go to A&E or present anywhere, or I don’t feel it would be appropriate so something like this would be actually ideal’* [Nurse and Academic].

### Perceived disadvantages of patient-owned DHTs

Participants were also invited to consider potential disadvantages of DHTs available to the public. Some of which have already been presented in previous sections of the findings, i.e. reliability concerns of both digital health devices and sources of information. Specifically, this section will contain disadvantages associated with DHTs that surfaced upon active prompting during interviews. Overall, these can be grouped as: a) anxiety and data obsession; b) patient autonomy and digital tech illiteracy; c) time and money wasting; d) data security and manufacturers’ agenda vs healthcare agenda.

a. Anxiety and data obsession

*i) ‘Our obsession with technology, you know, as a society, or inability to switch off from what’s going on – being over focused on numbers and data. Uhm, from a mental health perspective, you know, maybe I’m not enjoying my run, for example, because I’m looking to see what my watch is telling me about the speed that I’m going [and] I’ve missed the fact that there’s … a new species of bird next to me! Or the fact that the sun’s shining or there’s waves crushing … I’m actually focussed on whether or not my lap time is OK or whether or not I’ve beaten last week time. Uhm, I’m working towards numbers and focused on technology. […] you know, the other elements of life are becoming less important to me, if I’m focussed on numbers and data and self-tracking’* [GP and Academic Researcher].

*ii) I think it [self-tracking and patient-owned DHTs] would also lead to so much unnecessary anxiety and worry. In the population, generally, the levels of anxiety and, uhm, stress are exploding!’* [GP and Academic Lecturer].

b. Patient autonomy and digital tech illiteracy

*i) ‘The only disadvantage would be if, because of an app, somebody didn’t dial 999 or get the help that they needed […]* [Nurse and Academic].

*ii) ‘I think maybe […] the healthcare professional can’t monitor how it’s being used [patient’s DHT] or if it’s being used accurately, […] someone else could have been wearing it or they might have been using it wrong? So there’s that kind of checking by the healthcare professional is … you’re less able to do that. It’s down to how the patient uses it and whether they are using it correctly’* [Med Student].

*iii) ‘There is a reason why people go to med school for many years – there is only so much that digital apps can do. […] it probably gives you information, but it won’t help you process that information which is what a clinician can do so then [patients] rely and take the information coming from digital sources as ‘final’. Uhm, [I would suggest, instead] use that to question and have a discussion with your clinician’* [GH Expert].

c. Time and money wasting

*‘And the other concern [is] the influence on our health services in terms of resources. […] If somebody has an appointment with me to discuss the finer points of the read-out from their Apple watch, uhm, then there’s somebody else that day that is not being seen! […] the NHS is a limited resource […]. We don’t know if it’s good value for money … it may well be, I don’t know. But the cost and the resources taken to consult with, by enlarge, healthy people, about data that they are monitoring [pensive, sentence left open] … ’* [GP and Academic Researcher].

d. Data security and manufacturers’ agenda vs healthcare agenda

*i) ‘And the other thing is about our data. Patients may feel that they are being watched, that they are being recorded and, uhm, too much information about them is out there’ [Nurse].*

*ii) ‘Another [disadvantage] is the security of data, uhm, which is a considerable concern at the moment, especially with regards to health data. […] my device records when I sleep, how many steps I’ve done in a day … but, not everyone is going to be comfortable to necessarily share that information with their own device. And what happens to that information when it’s transmitted somewhere else? Would someone else have access to it? And how can it be used? And are businesses using this information as a method of currency?’* [Pharmacist].

*iii) ‘In my opinion, there are two different approaches to technologies, research and implementation. And there’s different individuals who are making technology and they all have slightly different agendas. So Apple, obviously, huge multi-national organisation, who are a commercial organisation – they want to make money out of products. Academic organisations are interested in research and what might help and what might work. […] And then, if they are effective, and not harmful, then implementing them. But there’s no onus on commercial companies to look at all the intended and unintended consequences before they implement. So you’ve got this miss-match between, sort of, research and implementation where lots of things are being implemented without efficacy testing. The opposite is true in academia – lots is being tested and not implemented!’* [GP and Academic Researcher].

### Perceived barriers towards implementation in healthcare practice

The significant lack of experience in dealing with patients and their DHTs, despite the evident increase in their popularity, led to the understanding that some factors may be hindering their incorporation in healthcare practice. As this became more evident, around midway through the data collection process of this study, participants were actively, and more systematically, asked to consider main/perceived barriers towards commercial DHTs implementation in healthcare practice. This resulted in the identification of a variety of different issues: a) patients’/HCPs’ digital literacy and mindsets (both often age-associated); b) costs, inequality, lack of resources and infrastructure; c) fast obsolescence of the tech; d) lack of evidence of effectiveness for clinical implementation.

a. Patients’/HCPs’ digital literacy and mindsets

*i) ‘People [patients] that are older, who are not familiar with the technology and they don’t know what FitBits are […], they might be feeling a bit hesitant to use [them]. So if their GP said “can you wear this throughout the week?” They might think “why[…]? I don’t know what to do with this”. So, I think, trying to overcome that barrier is really important’* [Med Student].

Conversely, one of the older participants did address the age/digital illiteracy issue, however, they did not see it to be nearly as extensive or ‘across the board’.

*ii) ‘Older people might wouldn’t embrace [DHTs]. But I do think, as well, that it does depend on the individual. We sort of tend to think that older people don’t use the technologies, but you know, sometimes they do! And they’re on Facebook and they’re doing Skype and using things so they might be willing [to adopt the tech more], so I think that [it’s more about] personality perhaps, than age!’* [Nurse and Academic].

*iii) ‘Staff resistance! [is a barrier]. […] It’s not in their culture – it’s in their life [as they use health apps themselves], but it’s not […] protocol. And they are not educated as well to do so – there are no guidelines’* [Nurse].

*iv) ‘I think health professionals are threatened sometimes by emerging technologies, uhm, and they sort of [say] “oh, don’t google that” or I’ve seen mugs with, uhm, “don’t confuse my medical degree with your Google search” and things like that. But actually, we probably ought to be focusing on the powerful benefit’* [GP and Academic Researcher].

*v) ‘Most of the older generation of physicians still see digital as something that is a fad, […] new and something not that reliable because they still believe that medicine is all person-oriented. Which is true to some extent! […] but digital technologies these days can make a lot of things streamlined and organised so … ’* [GH Expert].

b. Costs, inequality, lack of resources and infrastructure

*i) ‘Uhm, I think one of the main things for me would be the fact that these devices aren’t available to everyone? That sort of creates some inequality. So, if we’re going to use self-tracking, are we going to rely on people having their own devices such as an Apple Watch, Fitbit or whatever? And, not anybody is going to be able to afford that’* [Pharmacist].

Whilst inequality, in terms of access/ownership of DHT, did come up on some occasions when considering barriers, some participants also reflected on the fact that, in reality, now virtually everyone owns a mobile/digital device.

*ii) ‘And there might be a bit of inequality with respect to finances and data coverage and … but I think smartphone usage is permeated in society at every level and even in young people and children’* [GP and Academic Researcher].

*iii) ‘Nobody thought Instagram and Facebook would spread so fast in Africa but, look at it – everybody is on Instagram and Facebook. If you show the world the potential of any app, people will jump on to it. it’s just making sure that you have … that you eliminate the infrastructure barrier – that’s the key!’* [GH Expert].

And, indeed, infrastructure was another main barrier, both in terms of obsolete IT systems (developed world), as well as more basic, yet fundamental, aspects such as reliable internet and power (developing world).

*iv) My experience of IT in the NHS […] has been, uhm, very unfavourable. […] the IT systems, they are slow, they’re backward, they never work that well’* [GP and Academic Lecturer].

*v) ‘Uhm, not having good infrastructure and […] I mean, at the end of the day, for anything digital to work you need reliable internet, you need reliable power […], which are the basic things that need to be taken care of before … uhm, those are the main big barriers – infrastructure’* [GH Expert].

c. Fast obsolescence of the tech

*‘The other thing, I suppose, is that technology is developing so quickly – if we were to invest in the technology, how long is it going to be before it’s obsolete and needs to be replaced?’* [Pharmacist].

d. Lack of evidence of effectiveness for clinical implementation

*‘I think there’s a real need for some evidence in the area, particularly about what we do with abnormal readings [from patients’ own DHT devices] […] for example, if the patient had found a tiny short run of atrial fibrillation, for example, of their ECG reading, and they’ve been completely asymptomatic – is that equivalent to us finding it on a pulse check or an ECG in the practice?’* [GP and Academic Researcher].

### Suggested solutions to overcome disadvantages and barriers

Once barriers were identified, participants were asked to think of potential solutions to overcome them. These will not entirely match the previous section because, as mentioned, a more systematic approach to barrier/solution finding was not pursued from the start of data collection.

These solutions are grouped as follows: a) interventions on DHTs for official and trusted reliability; b) interventions on HCPs and patients to facilitate the understanding of rationale and purpose of DHTs; c) data security solutions; d) infrastructure solution.

a. Interventions on DHTs for official and trusted reliability

*‘There’s a massive gulf between what we’re using at the moment and what’s available [GHT-wise]. So I think the first step would be to give the clinicians better access and let us start to be using digital technology and then, maybe when we were getting more comfortable with it and happy that it was reliable and validated […], maybe […] patients could upload their input data sort of on NHS-type systems or something? That might be a better way around it, rather than patients individually holding these devices that nobody is calibrating or validating, and then giving us that data’* [GP and Academic Lecturer].

b. Interventions on HCPs and patients to facilitate the understanding of rationale and purpose of DHTs

*i) ‘Uhm … educate staff more. Maybe we should have presentations/lectures on digital health. Maybe the Uni could play a role to make us familiar. Because, not only the general population needs to be more educated, but also the healthcare professionals! And how to use the apps properly and which apps to use and how not to let, on the other hand, apps to take over our life!’* [Nurse].

*ii) ‘So maybe could do group sessions where healthcare professionals would be teaching individuals how to use the technology, and to understand why they [patients] are being monitored and what it’s for and what the benefit of that could be’* [Med Student].

The issue regarding data security concerns did not result in specific, concrete, solutions being found. However, it was considered that it may be more of a manufacturer duty to provide a safe online environment for data sharing.

c. Data security solutions

*i) ‘Data security is always going to be an issue. […] PII* [personally identifiable information] *has to be securely protected; it is always going to be an issue. But there are … I mean we have all of these apps, banking is done on apps, I do all my stock market on apps on my phone! So will there be security concerns? Yes, but whoever is developing the apps, I’m sure are capable of addressing those security concerns’* [GH Expert].

Similarly, to avoid potential liabilities upon data breaches, a participant added the following:

*ii) ‘I think confidentiality [issues] can be solved by somebody signing an agreement to be sent info via given channels’* [Nurse and Academic].

d. Infrastructure solutions

*‘Uhm I think the cell phone technologies are better suited in Sub-Saharan Africa than any other matters, because cell phones are widely used – everybody seems to have cell phones these days, especially smartphones. There’s reliable 3G in most parts of the world, uhm, it may not be 4G or 5G, but at least 3G is quite present. So taking the route of using cell phones to develop your digital technologies is the way to go rather than relying on any other digital sources. People are on their phones a lot! And keep it simple – anything complex that requires a lot of internet bandwidth is not going to be used at all because it won’t work – it has to be simple and provide the basic information in the native language’* [GH Expert].

### The future

Participants were also asked what they thought the future of patient-owned DHTs and cardiovascular healthcare may be like. The answers ranged from complete optimism about successful incorporation of the technologies in healthcare, to some level of scepticism.

*‘[in the future] you may be able to use technology to empower patients to look after their own health. And, also, relieve the burden on the NHS, because if you’re able to take that kind of bottom tear of people coming to the GP with problems that they could have looked online [or] on reliable apps and found out the answer to, then that will take away that percentage of people, [to] then free up GP appointments or any premises for people that actually need medical attention. So it’s definitely like a really bright prospect for the future, we just need better education!’* [Med Student].

‘*Well, I hope [in the future DHTs will be] very useful and very beneficial, time-saving, effective, uhm … partnership – more of a partnership approach to health; patients feeling autonomous with their health, getting their results and what they’re going to do with them. Uhm so that’s what I think I’m very positive about apps and how they might be used in healthcare*’ [Nurse and Academic].

*‘Uhm, not sure what it will look like. I guess it could look like two things. [Either] everybody having their watches and their phones and recording things, but nobody […] is really understanding why they’re doing that, or understanding what that means, [and interpreting] that data. And that is what I’m worried about – […] where would that work fall? Who’s gonna do that work? Uhm, or it could look like the NHS or clinicians have more control over that and we’re able to involve people in using apps or digital technology that is approved or validated or ones that have resources […] to help people with it […]. [And] I think* [meanwhile until the future of GHTs’ will be clearer, HCPs’ attitude should be] *one of healthy scepticism, recognising that this is promising. Recognising that it’s great that people are taking an interest in their health. Recognising the limitations and the fact that the evidence hasn’t caught up’* [GP and Academic Lecturer].

Both GPs (participants 003 and 005) also saw little value in current cardiovascular health self-tracking.

*‘And I think the big question that needs to be asked [is] – if you’re measuring pulse or BP or taking an ECG – why are you doing it? Why would you do that? […] We shouldn’t be blanket ECG-ing the whole population with Apple watches, because then we’ll just end up with lots of problems and lots of unnecessary over-investigation, over-diagnosis, completely moving away from the message of realistic medicine, which is the direction of travel that we know and most set out for us at the moment’* [GP and Academic Lecturer].

Nevertheless, participant 003 also considered the fact that, regardless of their value in healthcare practice, patient-owned DHTs are effectively generating an unprecedented amount of health/physiology-related data, which could potentially result in contribution to the current medical knowledge.

*‘Do we definitely understand the normal reference ranges [for heart rate]? And will our understanding of normal reference ranges change with big data from huge fitness companies? We might actually find something surprising about human beings and the way that their heart rate is!’* [GP and Academic Researcher].

## Discussion

We explored HCPs’ experiences of and views on commercially available DHTs, e.g. smartphones, smartwatches and health apps. The rationale was their perceived potential for integration into healthcare. We were especially interested in views on their value for CV disease. HCPs described current knowledge and experience and offered insights into barriers towards (and potential solutions for) constructive implementation of DHTs, where patients are already using them to self-manage and track aspects of their health.

### Experiences, knowledge and opinions

Overall, participants showed levels of personal interest in and familiarity with DHTs as users themselves, which is a potential bias in the sample (explored further below). Despite some scepticism regarding overall reliability, their use of DHTs is varied, covering different aspects of health: fitness, mindfulness and specific medical condition management. This is unsurprising, given the widely documented increase in these technologies’ popularity within society.^10^,^35^,^36^ However, their proliferation has not increased HCPs’ experiences in dealing with patients enquiring about them. This surprised them.

Reflecting their general lack of experience, participants felt their knowledge or preparedness to deal with patients enquiring about personal digital health devices and data is limited, because there are no clear and official guidelines for practice, or professional training available. This conflicts with the NHS push towards hosting a database of ‘approved apps’ among the vast array of choices available to anyone,^34^ suggesting awareness of, interest in and commitment to the growing phenomenon within the UK healthcare service. Given the current ‘early stages’ of DHTs, however, there could be a ‘lag’ between patients’ informal use of them, and subsequent more formal enquiries with a HCP, which in turn may justify the need for training and clearer guidelines for practice in the foreseeable future.

Participants, by contrast, detailed many experiences of patients presenting with information gathered online about their medical conditions and related treatments or courses of action. They often felt that patients focused on the ‘worst-case scenario’, causing unnecessary panic and sometimes even distrust in the healthcare provider. Patient use of the internet as a source of medical information has been a well-established phenomenon for decades now.^37–39^ A similar trend may have just begun with DHTs. ^3^,^10^,^40^

Despite lack of experience and concerns about professional engagement with commercial DHTs, opinions around their use in practice were positive in general. The predominant attitude was one of openness to discuss patients’ issues and concerns about DHTs’ outputs, albeit with caution, as little value was seen in overly frequent self-tracking of health-related parameters.

### Advantages, disadvantages and concerns

Several advantages and disadvantages associated with DHTs were identified. These were in line with current literature on the topic, such as behaviour change potential, patient autonomy and consequent time and resources implications.^41^,^42^ What is particularly interesting about our findings is how often the same aspect was seen as a potential advantage by some participants, and a disadvantage by others. An example of this is ‘patient autonomy’. Some participants believed that patient autonomy could lead to some level of self-care and self-monitoring by patients through DHT use, which may result in fewer GP appointments being booked, therefore some pressure relief on a national healthcare system known to be both financially and timewise burdened.^43^,^44^ Clearly, from this point of view, patient autonomy would be an advantage, saving time and resources. Other participants on the other hand saw the issue from a different angle as they felt that the implementation of commercial DHTs in routine healthcare could instead result in extra trouble-shooting (of any technological malfunction or patients’ inability to operate it) whose solution would become the HCPs’ responsibility, thus, extra time and resources being employed, i.e. a waste.

A concern that stood out most was that of patient data security. The two digital realms of the healthcare system vs. private companies, it was felt, are conceived with differing ultimate priorities; respectively – health care/promotion and profit. These concerns are quite legitimate, as precedents of data security issues are frequent in many personal and professional sectors, and the potential for patients’ data leakage has also been evaluated and documented, even among the NHS Apps Library.^45^,^46^ In this context of diverging agendas and interests between healthcare system and private digital companies, we also explored potential DHTs’ reliability issues and, as shown, concerns about them were prominent. Similarly, the overall trend of smartphone ownership might result in an increase of app use for health-related queries; unlike a simple and relatively anonymous web search, these apps tend to require *a priori*, and further generate, a significant amount of personal information which may well be of a sensitive/confidential nature,^47^ thus further justifying the participants’ concerns associated with data security.

### Barriers and solutions towards healthcare practice implementation

Among the barriers identified for constructive implementation of commercial DHTs in clinical settings, three of them require particular attention. First, inequality concerns – it is no surprise that DHTs, particularly wearables, are quite costly and not accessible to everyone. Moreover, those who cannot afford the technology due to financial restrictions, arguably, are the ones who could benefit the most from any form of health intervention, since poverty is a well-known indicator of poorer health outcomes.^48^,^49^ Unfortunately, providing patients with the necessary tech for health purposes under the NHS (or equivalent elsewhere) is financially unrealistic.

Second, in a developing context, such as Sub-Saharan Africa, DHTs also face a significant challenge for implementation in healthcare, given the significantly greater infrastructural barriers resulting in unreliable power and online access. The solution provided by the ‘GH Expert’ participant in this regard was quite simple and effective – anything wireless and battery operated could be a sufficient workaround since, contrary to general belief, ‘*everyone seems to be on their [smart]phones these days*’ and ‘*there is reliable 3G [internet coverage] in most parts of the world*’. Interestingly, this could also potentially solve the inequality concerns in parts of the developed world, as smartphone ownership has been progressively increasing and become nearly ubiquitous in (developed and developing) societies, more so than wearables.^50^

Third, some participants identified a significant lack of evidence of efficacy of DHTs for clinical practice and, consequently, a lack of clear guidelines to follow. This is strongly supported by recent findings in the literature that invite more in-depth research on the topic.^51^,^52^ Ideally, the evidence should come from pragmatic clinical trials, in order to assess the impact of interventions on objective outcome measures as rigorously and reliably as possible.^53^ Parallel (nested) qualitative assessment of the experiences of both HCPs and patients involved would also be undoubtably beneficial to gain valuable insight into potential barriers, pros and cons of DHT-based interventions.^54^

### Further considerations and recommendations

This study covered a variety of different themes within the current mHealth debate about DHTs and their implementation in healthcare. The original aim and objectives were to consider the phenomenon of DHTs increase in popularity with a focus on cardiovascular health and disease management and prevention, from HCPs’ points of view. However, the lack of experience of our participants in dealing with patients and their personal devices and data meant participants were more comfortable discussing the phenomenon in general terms. As a result, the cardiovascular aspect throughout data collection became more of an occasional topic, rather than a main component. This allowed the issue to be explored in broader terms, warranted by the realisation that patient-owned DHTs are clearly very much at early stages, much like the prospect of their constructive integration advocated in the literature.^13^,^55^

Given the ‘infancy’ of the phenomenon, many aspects of it are still fairly speculative, and this was evident in interviews with participants, which elicited differing views on the same aspects. As a methodological reflection, having multiple participants confronting each other at once in group discussions (compared with our individual interviews) might have produced further insights into the issue.^31^,^32^ FGs were part of the original plan for this study, however, participant recruitment practicalities determined that it was not possible to organise such sessions; perhaps a limitation. Another limitation might lie within the sample as its selection was non-random. Participants chose to volunteer their insights, a factor which could potentially have introduced some level of bias in the findings. Moreover, all but two of the nine participants were personal/professional contacts of the researchers, which might have positively influenced their decision to take part in the study.^56^ Most participants were early adopters of commercial DHTs themselves, hence personal awareness and interest might have played a role in their decision to volunteer and offer their insights, which may well differ from those of the general population of HCPs. This is further highlighted by the fact that one of the perceived barriers towards implementation was colleagues’ mindsets. Most participants were from (or at least practiced within) the UK, except for the ‘GH Expert’. This clearly restricts, though does not categorically preclude, the relevance of this study beyond the UK. The number of participants was also relatively small, therefore, while most themes recurred between participants, suggesting data saturation was achieved within our sample, we cannot be sure this is the case in the general population of HCPs, who may have even less experience with DHTs (personal or with patients). Nevertheless, the variety of both experts interviewed, and of relevant aspects touched on during the interviews, undoubtably enrich understanding, much like other qualitative work with just as many participants.^57^ Moreover, the concept of data saturation based on pre-set numbers, whilst valid in most instances, still remains a relative concept. This is demonstrated, for example, by Guest et al. who conducted a similar interview-based qualitative study in which data saturation was almost reached by the sixth interview out of sixty conducted in total, the bulk of which contributed very little new and relevant material.^58^,^59^ A strength of this study is the deeper level of insight acquired through participants, because of its qualitative nature and the adoption of semi-structured interviews which facilitated data generation around relevant themes and concepts.^60^,^61^ Some of these themes were in line with the recent literature, which also suggests that potential selection bias might have only minimally influenced our findings.

Given these findings, it appears premature to foresee the imminent implementation of DHTs in healthcare. However, this does seem to be the ‘general direction of travel’ and examples are: a) the already mentioned ‘NHS Apps Library’, ^34^ which suggest awareness of the popularity and the impact that commercial DHTs can have on patients; b) a recent partnership between Amazon Alexa® and the NHS,^62^ whereby answers to health-related questions from consumers in the UK will be sought from official NHS websites; c) the recent WHO guidelines for digital health interventions, advocating for the use of smartphone-based technologies as these are recognised to be well-spread in both developed and developing settings;^5^ and d) sustainable solutions aimed at providing the public with healthcare support via mainstream digital means, e.g. smartphones and apps, are clearly a main element on the agenda of HCPs involved in DHTs innovation, as gathered from the latest ‘Digital Catalyst’ event in Newcastle.^63^

At the time of conducting the research, it seemed premature according to our participants to focus on DHT experiences as most of the participants had not used DHTs with their CV patients. Early adopter, tech-enthusiast HCPs formed the majority of our sample and their interest in our study seemed to derive from their own personal experiences of DHTs rather than their experiences of working with CV patients. It was considered that DHTs were not mainstream enough yet, that there was little evidence for practice and that guidelines were in their infancy. These findings may help to explain why there is a dearth of qualitative studies of HCPs’ perceptions towards self-tracked CV health-related outputs as indicated in our introduction. Our work therefore contributes to the knowledge gap, however, this is only to report a lack of experience among our sample at this stage and cannot inform practice. We anticipate that the use of DHTs among CV patients will grow over the coming years and that the clinical implications will need to be researched. We also note that the impact of the COVID-19 pandemic, which began after the conclusion of our data collection and analysis, has led to speculation about increased reliance on DHTs and a rapid acceleration in the use of DHTs in clinical practice, including for CV patients, and therefore this unprecedented event will have policy and practice in unanticipated ways that need to be better understood in general and in relation to CV patients.^64–66^

Finally, further advancements are needed before integration of commercial DHTs can take place safely and constructively in healthcare. To achieve this, having explored HCPs’ perspectives and concerns on the issue, more research is warranted on several different aspects to: overcome the identified barriers; minimise the disadvantages; and assess the feasibility and effectiveness of these technologies in aiding universal healthcare provision, as in the UK’s NHS. A variety of different methodological approaches will be needed. For example, more focused and patient-oriented intervention studies assessing feasibility and effectiveness of commercial DHTs in managing specific conditions, e.g. cardiovascular diseases or diabetes, applying a rapid cycle evaluation methodology,^67^ may help address these gaps. Future studies should also aim to gather and take into account different patients’ views, experiences and considerations. Understandably, though, there are limitations towards rigorous study design and sustainable scaling up of interventions once deemed effective, e.g. resources availability, or lack thereof.^68^ Pursuing a practical partnership between healthcare and private digital technology companies, to establish common grounds and acceptable compromises between differing agendas, may be a valid starting point.

## Conclusions

Digital solutions undoubtably offer many opportunities for health care and promotion. Successful, safe and constructive integration of patient-owned DHTs into the digital healthcare systems available to HCPs is in its infancy and needs further work to assess and measure the effectiveness and, possibly, realise its potential in the near future. The challenges ahead are nevertheless substantial and further research should focus on addressing the disadvantages, minimising the identified barriers and finding long-term, safe and sustainable solutions for the implementation of technologies destined to increase in popularity and relevance.

**Appendix 1.** Data analysis framework.

Main theme	Code	Sub-code
Participant background(s)	Profession	CP
		GP/medical
		Nursing
		Allied/other
	Ethnicity	
	Experiences	
DH tech mentions	Wearables	
	Apps	
	Other	
Experience with patients/clients	Yes	
	Some	
	No	
Attitudes towards DH tech & patients	Positive	
	Negative	
Digital Health self-tracking advantages	Time/resources	
	Conversation	
	Patient autonomy	
	Behaviour change	
Digital Health self-tracking disadvantages	Time/resources	
	Conversation	
	Patient autonomy	Patient digital (il)literacy
		Data gaming
	Data security	Business data VS NHS data ownership and usage
	Inequality concerns	
	Data obsession	
Wearables and related outputs	Pros	
	Cons	
	Reliability	Accuracy
		Precision
		Error
		Calibration
Heath in the digital/online environment	Trusted sources	NHS Apps Library (approved apps)
	Social media	
	Other (e.g. blogs or specific websites)	
	Evidence-based information VS personal opinion	
Barriers towards implementation	Patient digital literacy	Age-associated digital illiteracy (−ve attitude from potential users who could benefit the most)
	Cost for patients (devices AND specific apps)	
	Cost for NHS	
	Software/hardware failure	
	Quick obsolescence of new tech	
	Patient compliance	
Solutions for implementation of DH tech in healthcare	Interventions on equipment	
	Interventions on HCPs	
	Interventions on patients	
	Other	
Future	Overall technological advancements and its consequences	
	Direction of future research	
	+ve aspects	
	−ve aspects	
Deviant themes	Data-secure translation services to overcome language barriers in a multicultural society such as the Scottish one	Barrier or opportunity? (e.g. for a new market/research area)
